# The effects of body dissatisfaction and depression levels on the dietary habits of university students in southern China during COVID-19

**DOI:** 10.3389/fnut.2023.1103724

**Published:** 2023-08-03

**Authors:** Chunmei Wu, Ming Hao, Xuesheng Liu, Di Yang, Bang Liu, Wenjing Yan, Qi Wang

**Affiliations:** ^1^School of Public Health and Health Management, Gannan Medical University, Ganzhou, China; ^2^Liaoning Province Center for Disease Control and Prevention, Shenyang, China; ^3^Key Laboratory of Prevention and Treatment of Cardiovascular and Cerebrovascular Diseases, Ministry of Education, Gannan Medical University, Ganzhou, China

**Keywords:** body dissatisfaction, depression, dietary habits, emotional eating behavior, university students, China

## Abstract

**Introduction:**

The novel coronavirus disease of 2019 has impacted people’s lives greatly. The spread of the pandemic has restricted many everyday social lives. Some studies have shown that strict risk control during the pandemic threatens people’s mental health and eating habits. University students vulnerable to mental health problems may have more prominent mental health and eating disorders during the pandemic. This study aims to elucidate the relationship between body dissatisfaction, depression, body mass index, and emotional eating among university students in the context of the pandemic in southern China. It provides a theoretical basis for developing future approaches to improve depression and emotional eating among university students.

**Methods:**

A total of 1,135 university students were recruited for the study. All participants completed anthropometric, body dissatisfaction, eating behavior, and depression level surveys.

**Results:**

The study finds that female students have higher levels of body dissatisfaction, depression, and emotional eating than male students. University students in the high body dissatisfaction group had higher levels of depression. Depression level (*β* = 0.33, *p* < 0.01), body dissatisfaction (*β* = 0.22, *p* < 0.01), sex (*β* = 0.16, *p* < 0.01), and income (*β* = 0.06, *p* < 0.01) were significant predictors of emotional eating. Fundamentally, this study highlights the impact of body dissatisfaction on depression and emotional eating.

**Discussion:**

The potential to improve depression and emotional eating among university students by improving their levels of body dissatisfaction was demonstrated.

## 1. Introduction

Since 2019, people’s lives have been affected to some extent by coronavirus disease of 2019 (COVID-19) in various countries (1). Many countries have adopted stricter quarantine policies to slow the spread of the pandemic (2). Some studies have shown that under strict quarantine, people’s eating behaviors have changed, and binge eating has increased (2, 3). A study with 1,097 adults in Poland revealed that more than half of the adult Poles snacked significantly more during the pandemic windfall than during the pre-pandemic period (4). The results of another study that included 1,047 people from Asia, Europe, and Africa as respondents showed that people’s food consumption patterns changed during the pandemic, with more snacking between main meals (5).

### 1.1. Mental health

Mental health issues, such as anxiety and depression, are thought to be important in changing people’s food consumption patterns during the pandemic (3). Studies have shown that emotional eaters believe that eating relieves negative emotions due to stress (6, 7). New crown pneumonia is spreading rapidly worldwide, causing mental health problems in many people (3). In addition to the fear of COVID-19 infection, forced reduction of social and isolation policies can lead to psychological problems such as depression (8). According to a systematic study of mental health findings in the general populations of eight countries, the prevalence of severe anxiety increased from 6.3% before the pandemic to 50.9%. The incidence of depression increased from 14.6% before the pandemic to 48.3% (9). A study conducted among Italian adults showed that nearly a quarter of people changed their eating habits because of higher anxiety levels (3).

### 1.2. Factors influencing dietary habits

Body image is the perception of one’s own body (10). It is closely related to many aspects of human functioning, including emotions, thoughts, behaviors, and relationships (11). Body dissatisfaction occurs when there is a discrepancy between the ideal and actual body shapes (12). It results in poor eating habits such as overeating (12, 13), and high levels of body image dissatisfaction may lead to depression (13, 14). High body mass index (BMI) may bring high risk of body dissatisfaction and dietary habits (12). Furthermore, body dissatisfaction is common among young people (13, 14), and under the pressure of the COVID-19 pandemic, body dissatisfaction may have a more serious negative impact on young people’s health (12).

### 1.3. The specificity of the university student population

University students experience changes in their social environments and face the challenge of moving from adolescence to adulthood (12). Because their parents do not supervise them, university students may find it difficult to maintain a healthy diet (15–17). They grow up, become independent adults as compared to children who were dependent on their parents while overcoming the pressures of academics, which makes them a vulnerable group for mental health problems (16–18). However, little research has been conducted on body dissatisfaction, depression, or emotional eating. Especially in the context of COVID-19, the physical and mental burden of university students may increase. Accordingly, this could further increase the likelihood of depression and emotional eating among university students.

### 1.4. Purpose

This study aims to explore the relationship between body dissatisfaction, depression levels, BMI and emotional eating behavior among university students in the context of the pandemic.

## 2. Materials and methods

### 2.1. Study participants

This study was conducted at a selected comprehensive university in Ganzhou City, Jiangxi Province, China. Participant recruitment information was disseminated on campus through posters put up in front of the dormitory building and leaflets distributed in study rooms. A total of 1,200 under graduate students were recruited and physically measured, and questionnaires were administered across the university between September and December 2021. As there were very few students older than 24 years, those who was older than 24 years were excluded from the study. Of these, 1,135 students (male: 555; female: 580) provided complete and valid data to be included in this study.

### 2.2. Body measurements

Height was measured using a height ruler with an accuracy (Seca 213, Germany) of 0.1 cm, and weight, body fat percentage, and muscle mass (0.1 kg precision) were measured using a body composition analyzer (Tanita BC-610, Japan). Body mass index (BMI; kg/m^2^) was calculated using height and weight; the BMI categories were underweight (BMI < 18.5), normal (18.5 ≤ BMI < 25), and overweight (BMI ≥ 25) (19).

### 2.3. Body type discontent

Sex-adapted silhouettes were used to assess body dissatisfaction among university students (20). The body type progression chart scores indicate a progression from the lowest obesity score to the highest muscular score. The lowest score was-7, and the highest score was 7. In this study, participants were asked to select their actual body silhouette (Current Silhouettes, CS) and their ideal body silhouette (Ideal Silhouettes, IS). The difference between the ideal body silhouette score and the actual body silhouette score is the level of body dissatisfaction. Allowing the definition of three categorized levels based on quartiles analyses: low dissatisfaction (|IS − CS | ≤ 1), medium dissatisfaction (2 ≤ |IS − CS| ≤ 4), high dissatisfaction (|IS − CS| ≥ 5) (20).

### 2.4. Emotional eating levels

The Chinese version of the Dutch Eating Behavior Questionnaire (21) was used to assess the level of emotional eating among university students. Emotional eating refers to the extent to which emotional factors influence college students. Here, emotion mainly refers to negative emotions and whether individuals will relieve their emotions by eating under the influence of negative emotions. For example, when individuals are depressed, they crave food. There were 13 questions, each with five options, corresponding to a score of 1–5. Higher scores indicated higher levels of emotional eating. This questionnaire is suitable for young Chinese adults and widely used in southern China (12).

### 2.5. Depression

The Chinese version of the Self-rating Depression Scale (SDS), widely used in China for young people, was used to assess the depression levels of university students (22). The scale contains 20 items divided into four scoring levels. The scores of each item are summed to obtain a total crude score, which is multiplied by 1.25 according to the Chinese norm to obtain a standard score; the higher the standard score, the more severe the symptoms. The standard score for evaluating depression is 53, with scores below 53 indicating no depression, 53–62 indicating mild depression, 63–72 indicating moderate depression, and 72 or more indicating severe depression. This scale has been verified to be suitable for young Chinese adults and widely used in southern China (12).

### 2.6. Data analysis methods

All items were tested for normality and skewness, and the data were within acceptable limits. We used a t-test to compare differences due to sex in BMI, body fat percentage, muscle mass, level of body dissatisfaction, level of depression, and score of emotional eating among university students. One-way ANOVA was used to compare differences in depression levels and emotional eating scores across body type groups, and the Tukey–Kramer test was used to compare two-by-two differences between groups. College students’ emotional eating scores were used as dependent variables in the multiple regression analyses, and BMI, depression level scores, body type dissatisfaction level scores, and monthly living expenses were used as predictor variables. A stepwise incremental approach with a threshold *p* value of 0.20 was chosen. All statistical analyses were performed using JMP version 16.0 J (SAS Institute Inc., Cary, NC, United States). Statistical significance was set at *p* < 0.05.

### 2.7. Sociodemographic characteristics

Data on the age, sex, and monthly living cost of the university students were collected through a questionnaire.

### 2.8. Sample size estimation

The sample size for the study was determined using the G*Power calculator 3.1.9.7 (Franz Faul et al., Universität Kiel, Germany, http://www.gpower.hhu.de/). Considering an α = 0.05, 1−*β* = 0.90, the number of tested predictors = 3 (SDS score, body dissatisfaction, and BMI), the number of covariates = 3 (age, sex, and monthly living cost), we calculated the sample size to be 33, 73, 528, respectively if the effect size f^2^ equaled to 0.35 (large), 0.15 (medium) and 0.02 (small). Furtherly, a 20% dropout rate was assumed, and the total number was estimated as 42–660. To make sure the power, we increased the sample size to 1,200, and the actual valid sample size was 1,135, which was much larger than the estimated size even if given a small effect size f^2^.

## 3. Results

### 3.1. Participant characteristics

The age of the participants ranged from 18 to 23 years (mean age: 18.8 ± 1.0 years; Table 1). Regarding BMI, 19% of the participants were overweight or obese (Table 1). Further, BMI, muscle mass, and obesity rates were higher in male students than in female students (*p* < 0.05). And body fat percentage, SDS, and emotional eating score were higher in female students than in male students (*p* < 0.05). Body dissatisfaction was present in both sexes (Table 1).

**Table 1:** Sample characteristics (*n* = 1,135).

	Mean ± SD or *n* (%)			*p*	Man (*n* = 555)	Woman (*n* = 580)	Total
Age (years)	18.8 ± 1.1	18.8 ± 1.0	18.8 ± 1.0	
BMI (kg/m^2^)	22.0 ± 3.6	21.3 ± 3.1	21.6 ± 3.4	<0.01
BMI category				
Underweight	80 (14)	96 (17)	176 (15)	
Normal	337 (61)	409 (71)	746 (66)	<0.01
Overweight/obesity	138 (25)	75 (13)	213 (19)	
Body fat percentage	16.1 ± 6.7	27.6 ± 5.9	22.0 ± 6.4	<0.01
Muscle mass (g)	50.5 ± 7.6	35.9 ± 4.0	43.0 ± 5.8	<0.01
SDS	44.9 ± 9.6	46.8 ± 8.8	45.8 ± 9.2	<0.01
Emotional eating score	22.5 ± 9.4	26.3 ± 9.8	24.4 ± 9.6	<0.01
Body dissatisfaction	2.8 ± 1.4	2.7 ± 1.3	2.7 ± 1.3	0.46
Low	164 (30)	184 (32)	348 (31)	0.51
Medium	273 (49)	286 (49)	559 (49)	
High	118 (21)	110 (19)	228 (20)	

BMI, body mass index. SDS, Self-rating Depression Scale. The significance of the differences between male and female students was determined by *t*-test (for quantitative variables) or by Pearson’s analyses (qualitative variables).

**Figure 1:**
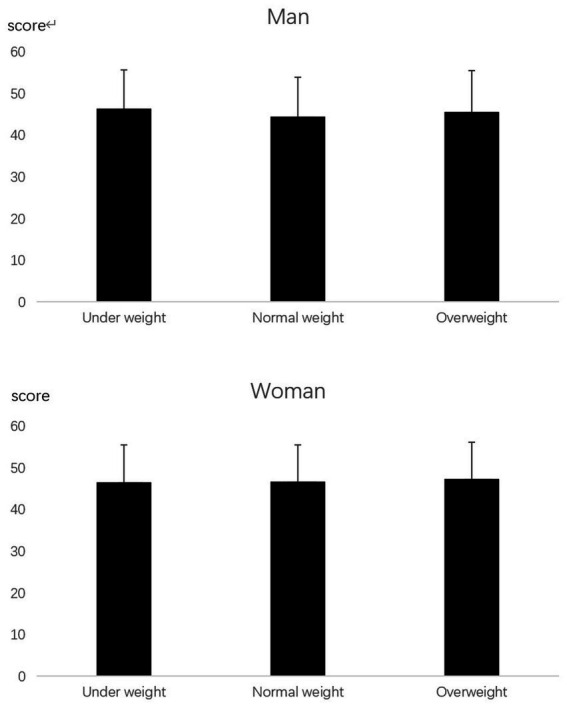
SDS scores of university students with different BMI categories.

**Figure 2:**
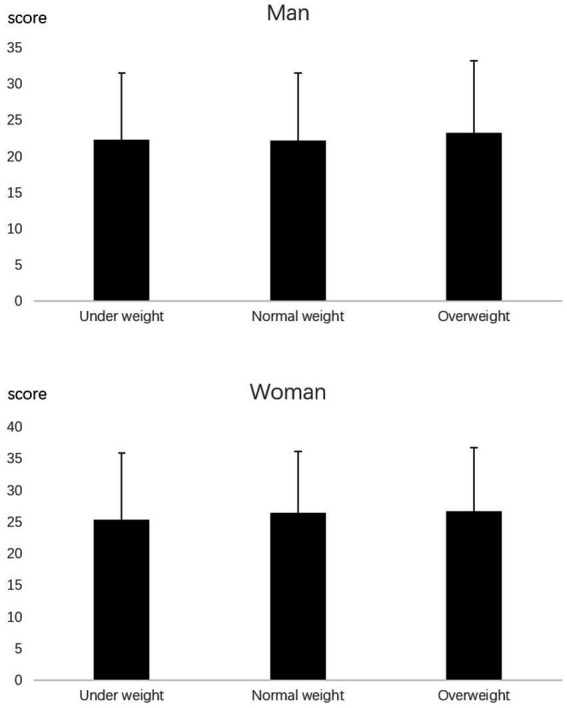
Emotional eating scores of university students with different BMI categories.

**Figure 3:**
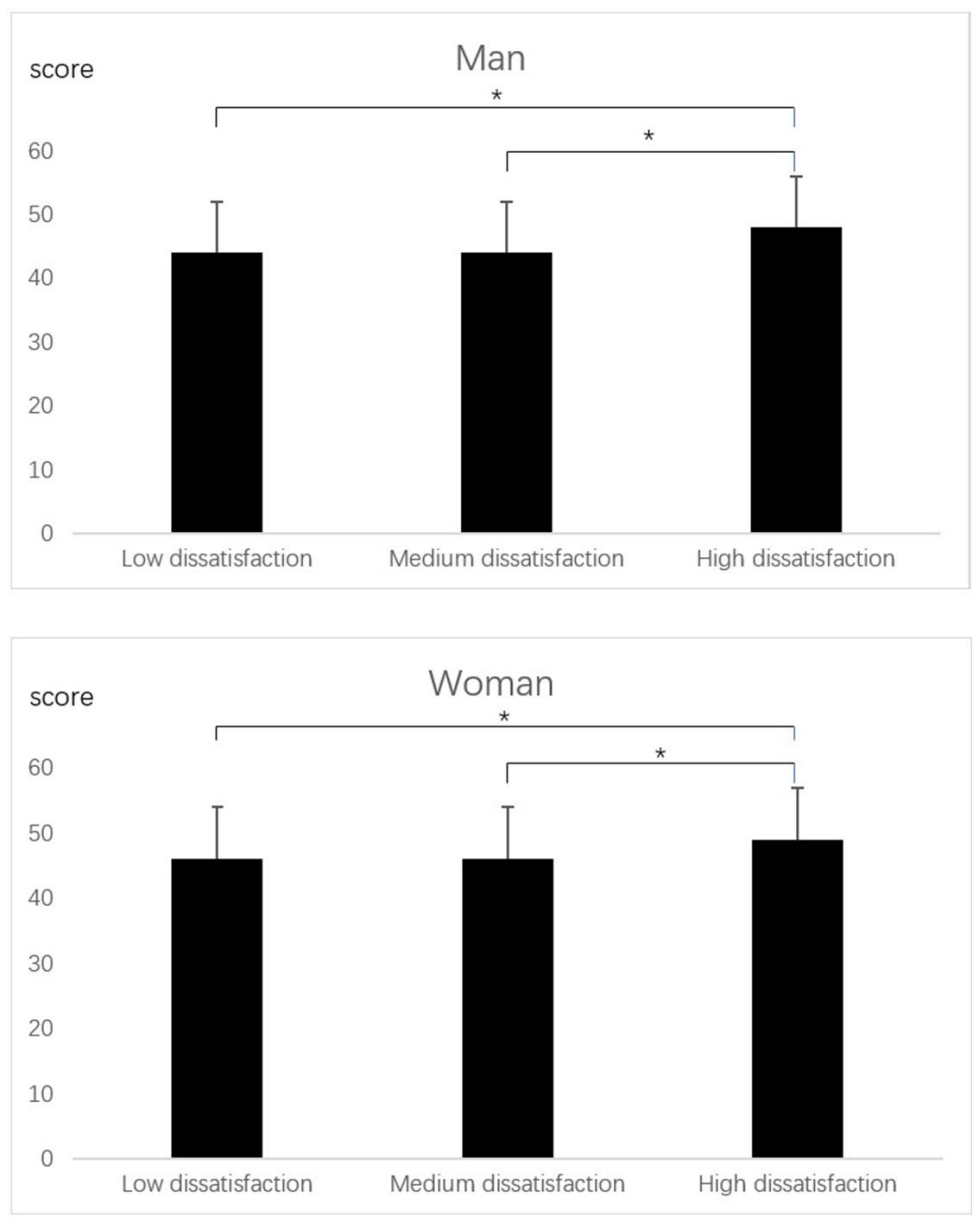
Depression scores of university students with different levels of body dissatisfaction. *Tukey, *p* < 0.05.

The mean emotional eating score for girls was 26.3, significantly higher than for boys (22.5; Table 1). There were no differences in depression levels or emotional eating scores between the body type groups (*p* > 0.05; Figures 1, 2). The emotional eating scores were significantly higher in the high body dissatisfaction group than in the moderate and low body dissatisfaction groups (Figure 3).

### 3.2. Factors influencing emotional eating

Table 2 shows the factors that influence emotional eating among university students. Levels of depression (*β* = 0.33, *p* < 0.01), body dissatisfaction (*β* = 0.22, *p* < 0.01), sex (*β* = 0.16, *p* < 0.01), and income (*β* = 0.06, *p* < 0.05) were significant predictors of emotional eating.

**Table 2:** Factors that contribute to emotional eating scores among university students.

	*β*	*t*	VIF	*p*
SDS	0.33	12.32	1.02	< 0.01
Body dissatisfaction	0.22	7.63	1.17	< 0.01
Sex	0.16	5.97	1.03	< 0.01
Income (/month)	0.06	2.13	1.01	< 0.05
BMI	−0.04	−1.37	1.18	NS

*R*^2^ = 0.21; *p* < 0.01; RMES = 8.8; *n* = 1,135.

## 4. Discussion

### 4.1. Emotional eating and depression levels among university students

#### 4.1.1. Emotional eating levels

Women were at a higher risk of developing health problems related to eating disorders. A study of Norwegian adults showed that emotional eating is more common in women (23). The results of the present study showed that among university students in southern China, women had higher levels of emotional eating than men (Table 1), supporting the findings of the prior study. This may be related to the physiological differences between men and women. Research suggests that the physiological differences between females and males may be reflected in emotions and behaviors, with girls likely to have increased negative emotions when faced with challenging and uncertain situations (24). Notably, emotional eating is considered an important way of reducing negative emotions, and eating disorders may be triggered or exacerbated by pandemics (25). Thus, sex differences in emotional eating levels may continue to be magnified by the influence of the pandemic.

#### 4.1.2. Interaction between depression and emotional eating

Emotional eating can lead to psychological distress and have negative health effects (26). A study of 1,865 Italian adults found that when considering multivariable models, women and individuals with emotional eating were more likely to report depression (26). These results support the findings of a previous study (Table 2); they demonstrated that higher levels of depression are related to higher emotional eating scores. At the same time, this effect was not unidirectional; results from a study of 24,968 inhabitants in Norway showed that people with psychological distress are four times more likely to experience emotional eating than the general population (23). A study of 365 Italian adults showed that increased emotional eating could lead to psychological problems such as depression and anxiety (27). Studies have shown that binge eaters and healthy people may regulate negative emotions through their diet (6, 7). Studies found that people who adopt emotional eating behaviors in the face of negative emotions do not reduce negative emotions during or after eating but instead increase their current negative emotions and are likely to adopt more aggressive eating behaviors in the face of negative emotions (28, 29). The interplay between depression and emotional eating may be a significant barrier to college students’ heart health during the pandemic.

### 4.2. Factors influencing emotional eating

#### 4.2.1. Depression levels

Women are more likely to suffer from depression than men (6). A survey of 286 university students from different ethnic groups in the United Kingdom found that women suffered from depression at twice the rate of men (30). Another survey of nearly 6,000 students in China showed that the prevalence of depression was 14% higher among female students than among male students (10%) (31). The results of this study showed that women scored higher levels of depression than men, supporting the findings of a previous study (Table 1). During COVID-19, women were more susceptible to the contagion of tension than men, as the normal range and duration of social activities were restricted, which had a more significant impact on the emotional problems of university students (32). A study of 385 Italian students aged 18–30 found that amidst the COVID-19 lockdown, 6% of the students develop more severe depressive symptoms (33). For the goal of “Zero COVID,” China witnessed the longest and strictest lockdowns, and have no targeted measures for improving mental health of university students. As the COVID-19 pandemic continues, with the increase of depression level, the difference in depression levels between men and women will likely continue to increase.

#### 4.2.2. Body mass index

BMI is considered a significant predictor of emotional eating (34, 35). Studies have highlighted the association between body size and eating habits (36, 37). However, in the present study, we did not find a relationship between body size and emotional eating or SDS score (Figures 1, 2). This suggests that, for present-day university students in southern China, emotional eating similarly affects food consumption in normal-weight and obese individuals. The intense pressure from the pandemic may be an important factor that distinguishes this study from previous research. The blockade brought about by the pandemic can impact emotional eating and change people’s eating habits (27). The results of this study reveal that emotional eating levels under the influence of the pandemic may not be related to body size.

#### 4.2.3. Body type discontent

Body image dissatisfaction is thought to be associated with poor eating habits such as dieting. A study of American high school students showed that students with higher levels of body dissatisfaction were more likely to have disordered eating behaviors than those with lower levels of body dissatisfaction (38). Similarly, a study of Dutch adolescents aged 12–16 showed that body image dissatisfaction might lead to disordered eating behaviors (39). The results of this study showed that higher levels of body dissatisfaction were associated with higher scores on emotional eating at university, supporting the findings of a previous study (Table 2).

It is important to note that because the weight loss effect of extreme eating behaviors may be short-lived, it usually results in weight regain and further increases body dissatisfaction (12, 40). Body image dissatisfaction may exacerbate depression in university students (Figure 3). Results from a study of 13,046 current university students in southern China showed that self-body awareness in early adulthood was associated with the onset of depression (13). A study with 160 Hispanic university students showed a positive correlation between levels of depression and levels of body dissatisfaction among university students (14). Therefore, improving the level of emotional eating among college students by improving their body image dissatisfaction should be considered.

### 4.3. Limitations

This study had several limitations. First, this study was conducted with only over 1,000 people and included only university students aged 18–23 years. In addition, because this was a cross-sectional study, no causal inferences could be drawn. Additionally, this study only examined university students living in southern China and is not representative of the national picture in general. The survey was carried out during the COVID-19 pandemic, with the strict policies “zero COVID” policy in China, and therefore, its results may not reflect the general relationship between body dissatisfaction, diet, and depression among university students. China witnessed the longest and strictest lockdowns of all, which might have skewed the results. Although a study has shown that these effects will disappear rapidly after the lifting of lockdown (33). Considering that the COVID-19 pandemic is still ongoing, the results of this study can provide a theoretical basis for formulating strategies to improve university students’ eating behavior and depression levels from the present to the near future. This study did not investigate which kind of food they ate/avoided during emotional eating, we wish to solve this problem in future research.

### 4.4. Conclusion

Depression and emotional eating were problems among university students in southern China in the context of the pandemic. Female students had higher depression levels and emotional eating scores than male students. This study highlighted the impact of body dissatisfaction on depression and emotional eating. The potential to improve depression and emotional eating among university students by improving their levels of body dissatisfaction has been demonstrated.

## Data availability statement

The original contributions presented in the study are included in the article/supplementary material, further inquiries can be directed to the corresponding author.

## Ethics statement

The studies involving human participants were reviewed and approved by ethical approval for the study was granted by the Gannan Medical University, China. The patients/participants provided their written informed consent to participate in this study.

## Author contributions

CW and MH: data collection, data analysis, manuscript writing, and funding acquisition. QW and XL: study design and data analysis. WY and DY: study design, data collection, data analysis, and manuscript writing. All authors contributed to the article and approved the submitted version.

## Funding

This study was supported by the Starting Research Fund of Gannan Medical University and Science and Technology Research Project of Education Department of Jiangxi Province (GJJ190814).

## Conflict of interest

The authors declare that the research was conducted in the absence of any commercial or financial relationships that could be construed as a potential conflict of interest.

## Publisher’s note

All claims expressed in this article are solely those of the authors and do not necessarily represent those of their affiliated organizations, or those of the publisher, the editors and the reviewers. Any product that may be evaluated in this article, or claim that may be made by its manufacturer, is not guaranteed or endorsed by the publisher.

## References

[1] Papandreou C Arija V Aretouli E Tsilidis KK Bulló M. Comparing eating behaviours, and symptoms of depression and anxiety between Spain and Greece during the COVID-19 outbreak: cross-sectional analysis of two different confinement strategies. Eur Eat Disord Rev. (2020) 28:836–46. doi: 10.1002/erv.2772, PMID: 32754986 PMC7436917

[2] Johnson AN Clockston RLM Fremling L Clark E Lundeberg P Mueller M. Changes in adults’ eating behaviors during the initial months of the COVID-19 pandemic: a narrative review. J Acad Nutr Diet. (2023):123, 144–194.e30. doi: 10.1016/j.jand.2022.08.132 PMC9444582 36075551

[3] Scarmozzino F Visioli F. Covid-19 and the subsequent lockdown modified dietary habits of almost half the population in an Italian sample. Foods. (2020) 9:675. doi: 10.3390/foods9050675, PMID: 32466106 PMC7278864

[4] Sidor A Rzymski P. Dietary choices and habits during COVID-19 lockdown: experience from Poland. Nutrients. (2020) 12:1657. doi: 10.3390/nu12061657, PMID: 32503173 PMC7352682

[5] Ammar A Brach M Trabelsi K Chtourou H Boukhris O Masmoudi L. Effects of COVID-19 home confinement on eating behaviour and physical activity: results of the ECLB-COVID19 international online survey. Nutrients. (2020) 12:1583. doi: 10.3390/nu12061583, PMID: 32481594 PMC7352706

[6] Macht M. How emotions affect eating: a five-way model. Appetite. (2008) 50:1–11. doi: 10.1016/j.appet.2007.07.002, PMID: 17707947

[7] Clum GA Rice JC Broussard M Johnson CC Webber LS. Associations between depressive symptoms, self-efficacy, eating styles, exercise and body mass index in women. J Behav Med. (2014) 37:577–86. doi: 10.1007/s10865-013-9526-5, PMID: 23934179

[8] Moreno C Wykes T Galderisi S Nordentoft M Crossley N Jones N. How mental health care should change as a consequence of the COVID-19 pandemic. Lancet Psychiatry. (2020) 7:813–24. doi: 10.1016/S2215-0366(20)30307-2, PMID: 32682460 PMC7365642

[9] Xiong J Lipsitz O Nasri F Lui LM Gill H Phan L. Impact of COVID-19 pandemic on mental health in the general population: a systematic review. J Affect Disord. (2020) 277:55–64. doi: 10.1016/j.jad.2020.08.001, PMID: 32799105 PMC7413844

[10] Schilder PM. The image and appearance of the human body: studies in the constructive energies of the psyche. New York: International Universities Press (1978).

[11] Pallan MJ Hiam LC Duda JL Adab P. Body image, body dissatisfaction and weight status in south Asian children: a cross-sectional study. BMC Public Health. (2011) 11:1–8. doi: 10.1186/1471-2458-11-21 21214956 PMC3025840

[12] Hao M Fang Y Yan W Gu J Hao Y Wu C. Relationship between body dissatisfaction, insufficient physical activity, and disordered eating behaviors among university students in southern China. BMC Public Health. (2022) 22:1–7. doi: 10.1186/s12889-022-14515-9 36352371 PMC9648036

[13] Zhang Y Liu B. Body weight perception and depressive symptoms in Chinese college students. Child Youth Serv Rev. (2021) 124:105969. doi: 10.1016/j.childyouth.2021.105969, PMID: 34682979

[14] Blow J Cooper TV. Predictors of body dissatisfaction in a Hispanic college student sample. Eat Behav. (2014) 15:1–4. doi: 10.1016/j.eatbeh.2013.10.010, PMID: 24411740

[15] Forestell CA Spaeth AM Kane SA. To eat or not to eat red meat. A closer look at the relationship between restrained eating and vegetarianism in college females. Appetite. (2012) 58:319–25. doi: 10.1016/j.appet.2011.10.015, PMID: 22079892

[16] Sandoz EK Boullion GQ Mallik D Hebert ER. Relative associations of body image avoidance constructs with eating disorder pathology in a large college student sample. Body Image. (2020) 34:242–8. doi: 10.1016/j.bodyim.2020.07.002, PMID: 32717626

[17] Muñoz-Rodríguez JR Luna-Castro J Ballesteros-Yáñez I Pérez-Ortiz JM Gómez-Romero FJ Redondo-Calvo FJ. Influence of biomedical education on health and eating habits of university students in Spain. Nutrition. (2021) 86:111181. doi: 10.1016/j.nut.2021.111181, PMID: 33618137

[18] Uri RC Wu YK Baker JH Munn-Chernoff MA. Eating disorder symptoms in Asian American college students. Eat Behav. (2021) 40:101458. doi: 10.1016/j.eatbeh.2020.101458, PMID: 33307468 PMC7906921

[19] WHO. Obesity: preventing and managing the global epidemic. Report of a WHO consultation. World Health Organization – technical report series. (2000) 894. 11234459

[20] Damasceno VO Vianna JM Novaes JS de Lima JP Fernandes HM Reis VM. Relationship between anthropometric variables and body image dissatisfaction among fitness center users. Rev Psicol Deporte. (2011) 20:367–82. doi: 10.1108/00483481111133345

[21] Wang YF Ha S Zauszniewski JA Ross R. Psychometric properties of the Chinese version of the Dutch eating behavior questionnaire in a sample of Taiwanese parents. Obes Res Clin Pract. (2018) 12:129–32. doi: 10.1016/j.orcp.2017.11.005, PMID: 29217141

[22] Zung WWK. A self-rating depression scale. Arch Gen Psychiatry. (1965) 12:63–70. doi: 10.1001/archpsyc.1965.01720310065008, PMID: 14221692

[23] Bemanian M Mæland S Blomhoff R Rabben ÅK Arnesen EK Skogen JC. Emotional eating in relation to worries and psychological distress amid the COVID-19 pandemic: a population-based survey on adults in Norway. Int J Env Res Pub Health. (2021) 18:130. doi: 10.3390/ijerph18010130 PMC7795972 33375442

[24] Hankin BL Abramson LY. Development of gender differences in depression: description and possible explanations. Ann Med. (1999) 31:372–9. doi: 10.3109/07853899908998794 10680851

[25] Güner Ö Aydın A. Determining the relationship between anxiety levels, stress coping styles, and emotional eating of women in the COVID-19 pandemic. Arch Psychiatr Nurs. (2022) 41:241–7. doi: 10.1016/j.apnu.2022.08.002, PMID: 36428056 PMC9385584

[26] Moro GL Bert F Catozzi D Scacchi A Siliquini R. Emotional eating and depression during the pandemic: quarant eat, an Italian nationwide survey. Nutrition. (2022) 103–104:111825. doi: 10.1016/j.nut.2022.111825 36183485

[27] Cecchetto C Aiello M Gentili C Ionta S Osimo SA. Increased emotional eating during COVID-19 associated with lockdown, psychological and social distress. Appetite. (2021) 160:105122. doi: 10.1016/j.appet.2021.105122, PMID: 33453336 PMC9755826

[28] Heatherton TF Baumeister RF. Binge eating as escape from self-awareness. Psychol Bull. (1991) 110:86. doi: 10.1037/0033-2909.110.1.86, PMID: 1891520

[29] Wallis DJ Hetherington MM. Emotions and eating. Self-reported and experimentally induced changes in food intake under stress. Appetite. (2009) 52:355–62. doi: 10.1016/j.appet.2008.11.007, PMID: 19071171

[30] Jenkins PE Ducker I Gooding R James M Rutter-Eley E. Anxiety and depression in a sample of UK college students: a study of prevalence, comorbidity, and quality of life. J Am Coll Heal. (2021) 69:813–9. doi: 10.1080/07448481.2019.1709474, PMID: 31995452

[31] Sun XJ Niu GF You ZQ Zhou ZK Tang Y. Gender, negative life events and coping on different stages of depression severity: a cross-sectional study among Chinese university students. J Affect Disord. (2017) 209:177–81. doi: 10.1016/j.jad.2016.11.025, PMID: 27923194

[32] Chang J Yuan Y Wang D. Mental health status and its influencing factors among college students during the epidemic of COVID-19. Nan Fang Yi Ke Da Xue Xue Bao. (2020) 2020:171–6. doi: 10.12122/j.issn.1673-4254.2020.02.02 PMC7086131 32376528

[33] Meda N Pardini S Slongo I Bodini L Zordan MA Rigobello P. Students’ mental health problems before, during, and after COVID-19 lockdown in Italy. J Psychiatr Res. (2021) 134:69–77. doi: 10.1016/j.jpsychires.2020.12.045, PMID: 33360865

[34] Nyklíček I Vingerhoets AD Zeelenberg M. Emotion regulation and well-being: a view from different angles In: Nyklíček I Vingerhoets A Zeelenberg M, editors. Emotion regulation and well-being. New York: Springer (2011). 1–9.

[35] Konttinen H Männistö S Sarlio-Lähteenkorva S Silventoinen K Haukkala A. Emotional eating, depressive symptoms, and self-reported food consumption. A population-based study. Appetite. (2010) 54:473–9. doi: 10.1016/j.appet.2010.01.014, PMID: 20138944

[36] Ozier AD Kendrick OW Leeper JD Knol LL Perko M Burnham J. Overweight and obesity are associated with emotion-and stress-related eating as measured by the eating and appraisal due to emotions and stress questionnaire. J Am Diet Assoc. (2008) 108:49–56. doi: 10.1016/j.jada.2007.10.011 18155989

[37] Sevinçer GM Konuk N. Emosyonel yeme. J Mood Disor. (2013) 3:171–8. doi: 10.5455/jmood.20130926052526

[38] Valerie FH. High prevalence of abnormal eating and weight control practices among U.S. high-school students. Eat Behav. (2004) 5:325–36. doi: 10.1016/j.eatbeh.2004.04.003, PMID: 15488447

[39] Muris P Meesters C van de Blom W Mayer B. Biological, psychological, and sociocultural correlates of body change strategies and eating problems in adolescent boys and girls. Eat Behav. (2005) 6:11–22. doi: 10.1016/j.eatbeh.2004.03.002, PMID: 15567107

[40] Hao M Han W Yamauchi T. Short-term and long-term effects of a combined intervention of rope skipping and nutrition education for overweight children in Northeast China. Asia Pac J Public Health. (2019) 31:348–58. doi: 10.1177/1010539519848275, PMID: 31091980

